# Case Report: Solving a decade-long paroxysmal hypertension mystery: bladder paraganglioma

**DOI:** 10.3389/fonc.2025.1624336

**Published:** 2025-09-09

**Authors:** Xingchen Li, Wuwu Ding, Dong Lv, Hong Li

**Affiliations:** ^1^ Department of Radiology, People’s Hospital of Deyang City, Deyang, Sichuan, China; ^2^ Department of Pathology, People’s Hospital of Deyang City, Deyang, Sichuan, China; ^3^ Department of Urology, People’s Hospital of Deyang City, Deyang, Sichuan, China

**Keywords:** bladder paraganglioma, neuroendocrine tumor, paroxysmal hypertension, ambulatory blood pressure monitoring, partial cystectomy

## Abstract

Bladder paraganglioma (BPGL) is an exceptionally rare neuroendocrine tumor whose diagnosis is often delayed or missed owing to its infrequent occurrence and variable clinical manifestations. We report a case of a 72-year-old female with a decade of unexplained paroxysmal hypertension, in which the diagnosis was ultimately prompted by an incidentally discovered bladder mass on ultrasonography. Crucially, 24-hour ambulatory blood pressure monitoring revealed micturition-triggered hypertensive crises, guiding subsequent confirmation through elevated post-voiding plasma-free metanephrines. The patient underwent successful laparoscopic partial cystectomy following careful preoperative alpha-adrenergic blockade, achieving complete symptom resolution postoperatively. This report underscores the diagnostic challenges inherent to BPGL, particularly the inconsistent presentation of classic micturition-related symptoms that can obscure the underlying trigger, emphasizing the utility of detailed history taking combined with targeted investigations, such as ABPM, to establish the diagnosis when initial catecholamine screenings may be unrevealing. Furthermore, this case reinforces the critical importance of meticulous preoperative preparation, multidisciplinary surgical management, and the necessity for considering genetic counseling and lifelong surveillance, given the potential for malignancy and hereditary predisposition associated with this condition.

## Introduction

According to the World Health Organization (WHO) classification system, pheochromocytomas and paragangliomas (PPGLs) are distinguished based on tumor location; those occurring in the adrenal gland are termed pheochromocytomas, while those occurring outside the adrenal gland are designated as PPGLs because they are histologically indistinguishable (1). Extra-adrenal PPGLs are inherently uncommon, accounting for approximately 10% of all catecholamine-producing tumors (2).

Bladder paraganglioma (BPGL) is an even rarer entity, accounting for less than 1% of all PPGLs and less than 0.05% of all primary bladder tumors (3). While typical symptoms, such as severe high blood pressure, palpitations, headache, heavy sweating, or fainting associated with urination, can alert doctors to BPGL, this only occurs in some patients. Many others present with mild, atypical symptoms or have no symptoms at all (4). This extreme rarity, coupled with variability in clinical presentation, makes BPGL highly susceptible to being missed or misdiagnosed. Early and accurate diagnoses significantly affect the prognosis of patients with BPGL. Delay or misdiagnosis can lead to inappropriate surgical choices, potentially triggering severe cardiovascular complications.

Herein, we report a case of BPGL that was diagnosed after more than a decade of delay. The patient had long-standing paroxysmal hypertension with dizziness and palpitations, but the cause remained unknown despite many doctor visits. The diagnosis began to emerge only after the bladder mass was incidentally discovered on ultrasonography. This report details the case’s diagnosis and treatment, reviews the literature, and discusses BPGL’s features, diagnostic challenges, treatment, and follow-up of BPGL. We aimed to improve the clinical awareness and management of this rare condition.

## Case presentation

**Figure 1:**
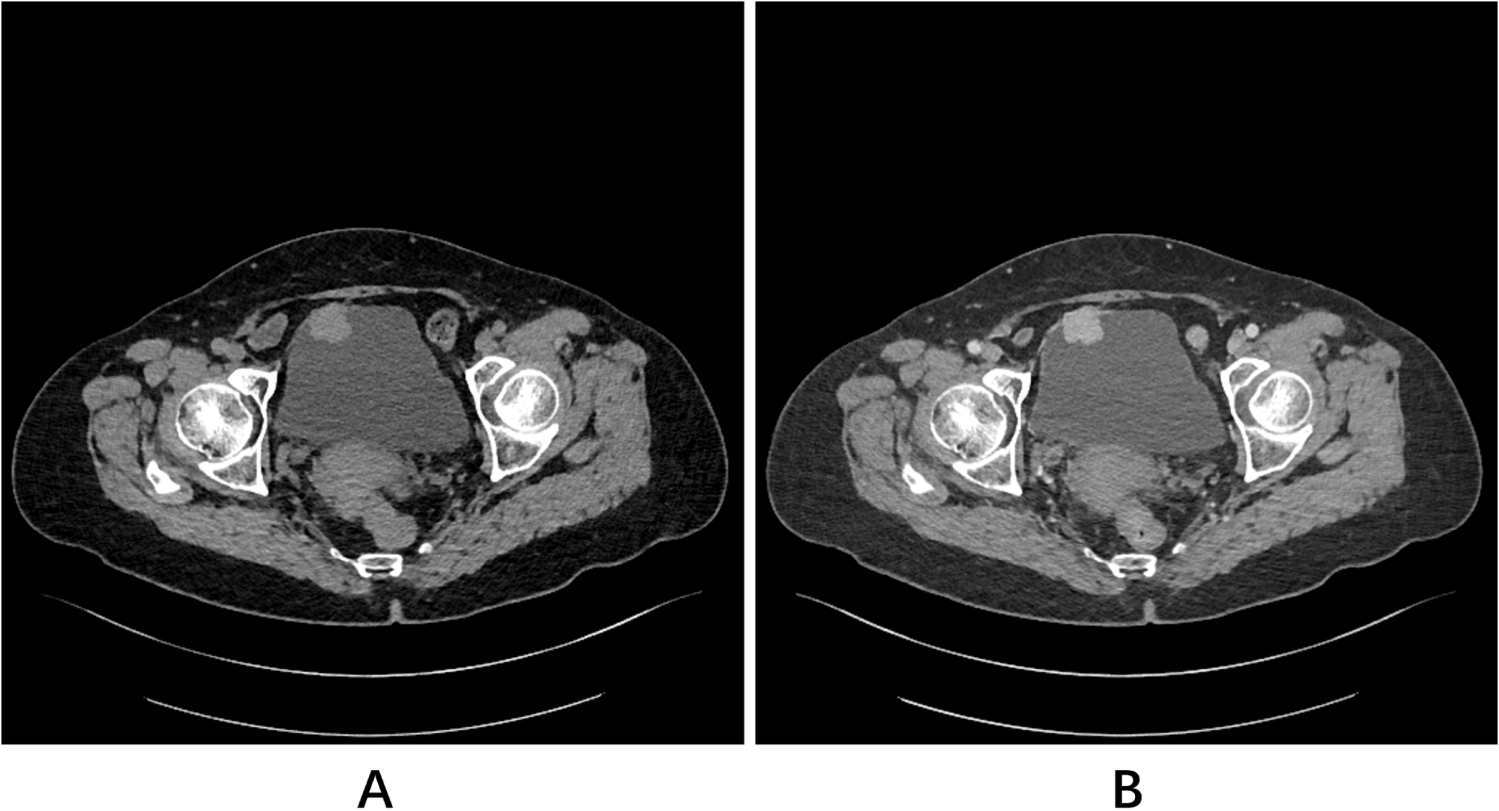
Pelvic CT scans. **(A)** Plain scan and **(B)** contrast-enhanced scan revealed a homogeneously and markedly enhancing mass located in the anterior dome of the bladder.

A 72-year-old woman presented with right flank pain, prompting an ultrasound that incidentally detected a bladder mass. Detailed imaging with contrast-enhanced computed tomography (CT) of the abdomen and pelvis confirmed the presence of a 2.6 × 2.2 cm, well-defined mass on the anterior dome of the bladder, located away from both ureteric orifices and exhibiting uniform and marked enhancement (**Figures 1A, B**). The scan also provided an explanation for her presenting symptom, revealing a 0.6 × 0.3 cm stone in the intramural segment of the right ureter, associated with mild proximal dilation. The left ureter and both kidneys were otherwise unremarkable.

Reviewing her medical history, it was noted that the patient had experienced episodes of paroxysmal hypertension for over a decade. During these spells, her systolic blood pressure surged above 200 mmHg, often causing dizziness and palpitations. Between these occurrences, her baseline blood pressure typically remained within the normal range or was somewhat low. She had previously visited other medical centers on multiple occasions seeking help for these symptoms. Evaluations during those prior visits involved investigations of adrenal gland disorders, including pheochromocytoma, but did not reveal any positive findings. She reported no history of blood in her urine, increased urinary frequency, or urgency.

**Figure 2:**
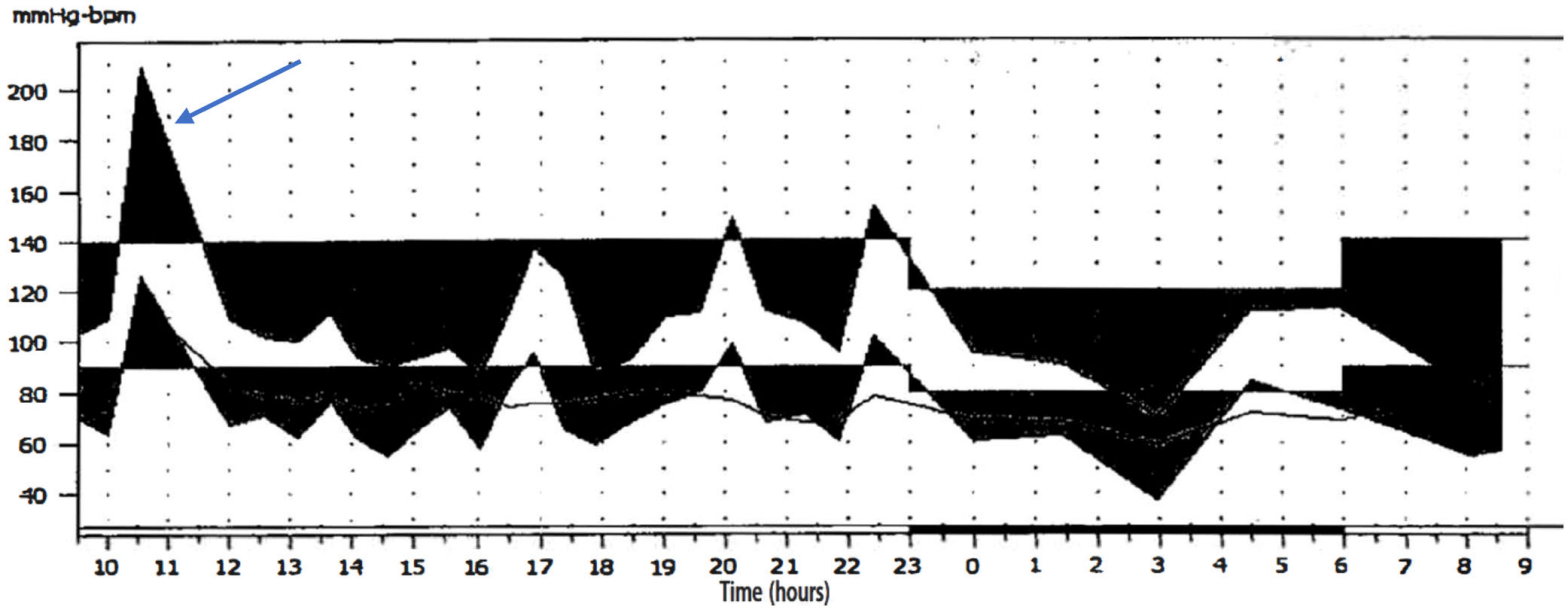
24-hour ABPM revealed a significant surge in blood pressure (> 200/120 mmHg) (arrow). Other readings were generally unremarkable.

On physical examination at the time of admission, her blood pressure was 86/58 mmHg. Given the significant blood pressure lability and indeterminate nature of the bladder mass, 24-hour ambulatory blood pressure monitoring (ABPM) was performed to investigate a potential association. The monitoring successfully captured an episode of acute hypertensive crisis (peak blood pressure >200/120 mmHg), which occurred immediately following micturition, with readings otherwise normal (**Figure 2**).

Based on the constellation of findings, specifically the characteristic clinical presentation of micturition-induced paroxysmal hypertension, a long-standing history of blood pressure volatility, and the presence of a hypervascular bladder mass, BPGL, was strongly suspected. To verify this hypothesis, we subsequently performed a post-voiding plasma-free metanephrine assay. The measurement showed a plasma metanephrine level greater than 20.56 nmol/L (whereas the normal range was 0.6 - 0.9 nmol/L), a finding that supports BPGL diagnosis. Given that the contrast-enhanced abdominal CT, along with a routine non-contrast chest CT, revealed no other suspicious lesions in common PPGL sites, and considering the tumor’s small size and the absence of a relevant family history, the patient was deemed to be at a low overall risk for multifocal disease. After a shared decision-making process that weighed the limited additional diagnostic yield of functional imaging against its significant cost, the patient opted to forgo studies like Ga-68 DOTATATE PET/CT.

After ruling out surgical contraindications, we proceeded with eight days of preoperative preparation for the patient. This regimen involved an oral alpha-adrenergic blocker (phenoxybenzamine) coupled with adequate volume expansion. The patient subsequently underwent laparoscopic partial cystectomy under general anesthesia. Intraoperatively, during tumor resection, the patient’s blood pressure increased to 220/110 mmHg. Prompt pharmacological intervention by the anesthesia team stabilized her blood pressure. The tumor was ultimately resected completely.

**Figure 3:**
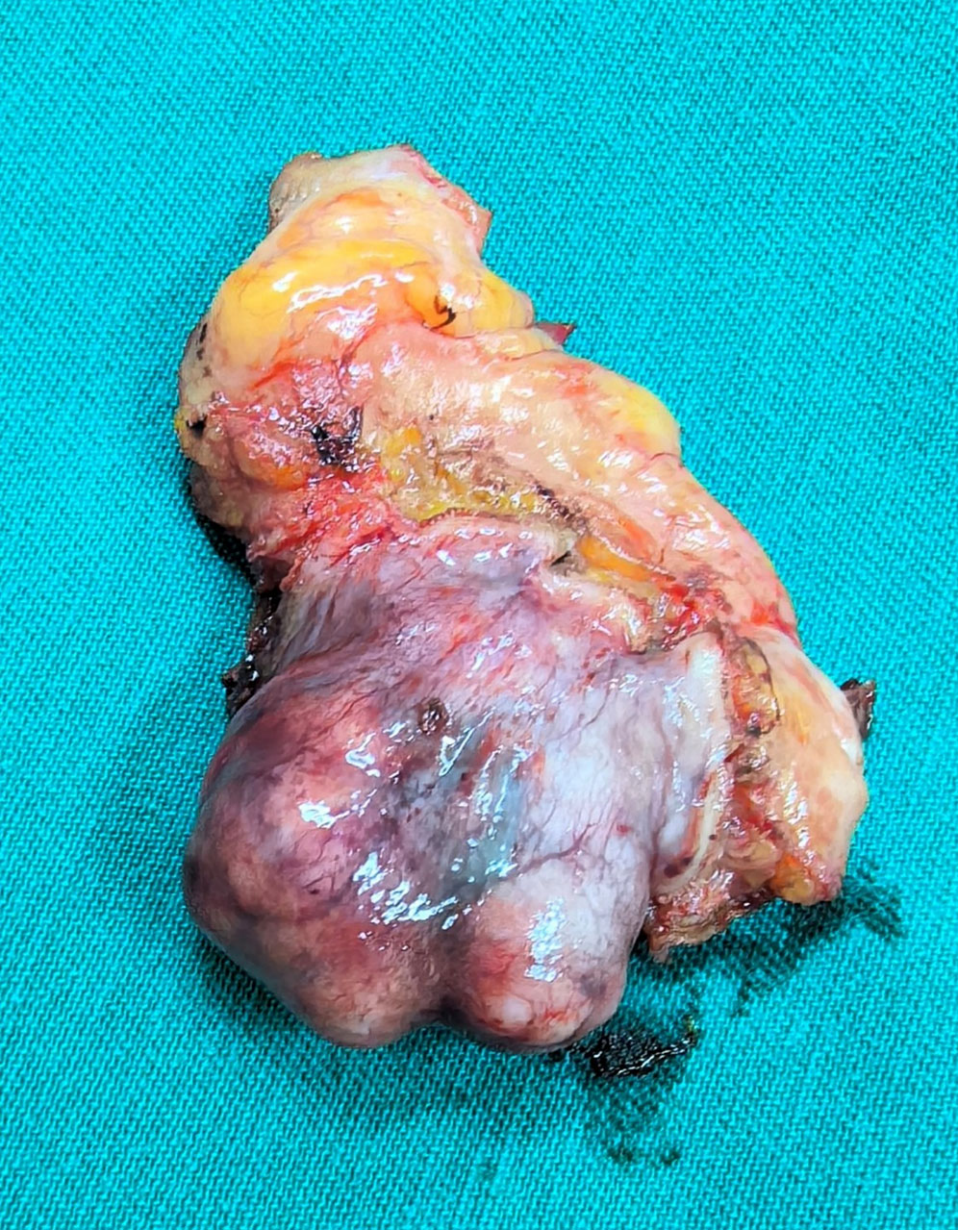
Postoperative gross specimen showed the tumor to be well-encapsulated.

**Figure 4:**
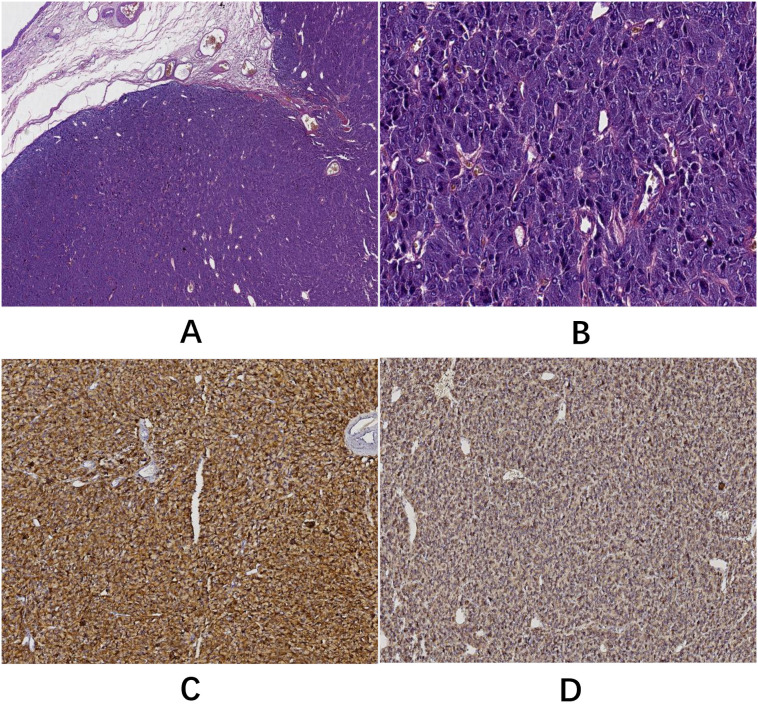
**(A)** H&E staining (5× objective): the tumor displayed clear borders and a nodular growth pattern. **(B)** H&E staining (40× objective): neoplastic cells were organized into characteristic nests (Zellballen) by abundant capillaries with occasional pseudorosette-like arrangements. Cells resembling normal chromaffin cells showed marked pleomorphism. Mitotic activity was not observed. **(C)** Immunohistochemical staining (EnVision method) demonstrated cytoplasmic positivity for chromogranin A (CgA). **(D)** Immunohistochemical staining (EnVision method) showed cytoplasmic positivity for synaptophysin (Syn).

Postoperative gross examination of the specimen revealed a well-encapsulated mass (**Figure 3**). Histopathological examination, supplemented with immunohistochemical staining, confirmed the diagnosis of BPGL (**Figure 4**). The patient’s postoperative recovery was uneventful. She declined further genetic testing. While she reported complete symptom resolution and stable blood pressure at her 3-month follow-up, a lifelong surveillance plan has been instituted due to the inherent risk of recurrence. This protocol consists of annual biochemical screening (measurement of plasma or urinary catecholamines and their metabolites), supplemented by yearly contrast-enhanced CT or MRI of the surgical site for at least the initial three years to monitor for local recurrence or new tumor development.

## Discussion

BPGL is a rare neuroendocrine tumor arising from paraganglia within the bladder wall (5). As demonstrated in the present case, the diagnosis of BPGL often presents significant challenges. Its rarity, variable clinical presentation, and similarity to more common bladder conditions can contribute to diagnostic delay or misdiagnosis.

First, the extreme rarity of BPGL presents a major diagnostic challenge. The overall prevalence of pheochromocytoma and PPGL is low, estimated at just 0.2 - 0.6% in people with hypertension (6), and cases specifically involving BPGL constitute an extremely small subset. A Danish population-based study estimated its annual incidence rate to be merely 0.16 cases per million person-years (5). This infrequency means that clinicians, even specialists such as urologists and endocrinologists, may encounter few, if any, cases throughout their professional careers, consequently lowering the index of suspicion for this diagnosis.

In recent years, the widespread adoption of advanced imaging modalities has led to a significant increase in the proportion of incidentally discovered PPGLs (7). The present case exemplifies this trend as the patient’s bladder mass was detected incidentally during an ultrasound examination performed for flank pain. This underscores the importance of evaluating patients with paroxysmal hypertension, considering the possibility of neuroendocrine tumors in less common locations beyond typical adrenal sites, even after initial negative workups for adrenal pathology. Expanding the scope of diagnostic imaging may be warranted in selected cases to increase the likelihood of identifying these rare lesions.

The marked heterogeneity in the clinical presentation of BPGL represents another significant factor contributing to diagnostic difficulty. The hallmark sign is the ‘micturition attack,’ characterized by hypertensive episodes linked to urination, which often include symptoms like headache, palpitations, diaphoresis, or syncope (4). This phenomenon is attributed to the abrupt, massive release of catecholamines triggered by bladder wall contraction or direct tumor compression during voiding (8). However, the diagnosis of BPGL is frequently complicated by non-specific presentations such as hematuria, persistent hypertension, or paroxysmal hypertension unrelated to voiding, with some patients being entirely asymptomatic (9, 10). This challenge is underscored by research from Li et al., which found that while hypertension and sympathetic symptoms were common in the group diagnosed with BPGL preoperatively, hematuria was predominant in those identified only after surgery, suggesting that non-specific symptoms readily lead to misdiagnosis and improper treatment (11).

Moreover, even when classic micturition-induced symptoms are present, their pattern is notoriously inconsistent, emerging during, immediately after, or several minutes following urination (8, 12, 13). In our case, despite 24-hour monitoring confirming a link between hypertension and urination, only a single hypertensive event was observed. This demonstrates that symptoms do not manifest with every micturition. This variability likely stems from inconsistent catecholamine secretion, potentially influenced by factors such as the intrinsic secretory activity of the tumor and the specific nature or intensity of the stimulus. Consequently, this erratic pattern of symptom onset can prevent patients from recognizing the connection between their symptoms and urination—a critical clinical insight that appears to be underemphasized in the existing literature. This wide spectrum of clinical presentations mandates that clinicians maintain BPGL within their differential diagnoses when encountering patients with unexplained hypertension (particularly paroxysmal patterns), hematuria, or an incidental bladder mass.

Imaging studies are essential to locate BPGL. Computed tomography (CT) is typically the first choice because it provides excellent spatial details of the chest, abdomen, and pelvis (6). Although magnetic resonance imaging (MRI) offers advantages in specific scenarios, such as detecting skull base and neck PPGLs, CT generally demonstrates higher sensitivity for localizing pheochromocytomas and PPGLs (6). However, MRI may provide superior details regarding tumor characterization and assessment of local invasion (14).

In the present case, contrast-enhanced CT revealed a well-defined mass on the anterior dome of the bladder protruding into the lumen and exhibiting uniform, marked enhancement, which is consistent with the typical imaging characteristics of PPGL described in the literature (14, 15). However, relying solely on the anatomical appearance observed on CT or MRI is often insufficient to confidently distinguish BPGL from other bladder tumors, particularly urothelial carcinoma (16). This limitation underscores the need for a thorough consideration of the differential diagnosis.

Given the hypervascular nature of the mass in our case, the differential diagnosis primarily involves other enhancing bladder lesions. The most critical distinction is from urothelial carcinoma, the predominant bladder malignancy, which clinically often presents with gross hematuria. On imaging, while it can be hypervascular, its enhancement is typically less avid and more heterogeneous compared to the intense, uniform uptake characteristic of paraganglioma (17, 18). Furthermore, several other entities can mimic this imaging phenotype. Benign hemangiomas can show avid arterial enhancement, though the presence of phleboliths may offer a diagnostic clue. Rare tumors like primary extramedullary plasmacytoma may also present with marked homogeneous enhancement, creating significant overlap on imaging alone (19). In contrast, other mesenchymal malignancies such as leiomyosarcoma or rhabdomyosarcoma usually appear as larger, infiltrative masses with heterogeneous enhancement patterns (19). Ultimately, imaging findings alone are often non-specific. The definitive differentiating factor for bladder paraganglioma is its functional nature. Therefore, correlating imaging with clinical signs of catecholamine excess and confirming with targeted biochemical testing remains the indispensable pathway to an accurate diagnosis.

Building on this need to assess the tumor’s functional nature, Ga-68 DOTATATE PET/CT is a widely accepted imaging modality for neuroendocrine tumors, demonstrating excellent accuracy in detecting PPGLs due to their high expression of somatostatin receptors (SSTRs) (20). Its application is generally recommended for staging patients at higher risk of multifocal or metastatic disease, such as those with large tumors (>10 cm), an extra-adrenal location, or a hereditary predisposition (21). While our patient’s case fell into the extra-adrenal category, a risk-stratified approach was adopted. Several mitigating factors were considered: the tumor’s small size suggested a low metastatic potential; the patient lacked any family history or signs of a hereditary syndrome; and importantly, concurrent CT imaging of the chest and abdomen had already ruled out other suspicious lesions in common PPGL sites. In this context, and following a shared decision-making process that also considered the patient’s economic situation, we proceeded directly to surgery. This tailored preoperative pathway, however, underscores the critical importance of the rigorous long-term surveillance we instituted postoperatively to diligently monitor for any potential recurrence or metachronous disease.

Measuring plasma or urinary catecholamines and their metabolites provides crucial biochemical markers for the diagnosis of pheochromocytoma and PPGL (7). However, applying this testing effectively presents significant challenges in the context of BPGL. This difficulty arises primarily because catecholamine secretion is often paroxysmal and frequently triggered only by specific stimuli such as micturition. Consequently, levels measured in resting-state samples or from randomly collected 24-hour urine or plasma specimens may fall entirely within the normal range (13, 22). Furthermore, in patients with BPGL, circulating catecholamine levels might have already normalized by the time significant hypertension became clinically apparent. This phenomenon could stem from physiological delays involving receptor activation and subsequent vascular smooth muscle contraction (23). The negative results from our patient’s previous extensive workups for adrenal pathology may be explained by these factors.

This experience suggests that, for paragangliomas in unusual locations, exploring the characteristic symptom triggers might have a greater initial diagnostic value than relying solely on biochemical tests. As illustrated by the diagnostic process in this case, biochemical assays are best reserved for confirming a diagnosis once clinical suspicion is aroused. Critically, for paragangliomas with such intermittent secretion, once a potential trigger is identified, biochemical tests must be performed shortly after the triggering activity. This approach significantly boosts the likelihood of capturing a positive result, given that plasma catecholamine levels surge rapidly after the stimulus and are quickly cleared from circulation (23).

Therefore, drawing from the experience of this case, we propose a more effective diagnostic workflow for patients presenting with unexplained paroxysmal hypertension or symptoms of sympathetic hyperactivity. After the routine exclusion of adrenal diseases, it is crucial to promptly employ ancillary investigations, such as ABPM or AECG, to identify specific triggers. Then, guided by these triggers, we can choose the perfect moment for biochemical testing. Following this process can substantially improve the chances of detecting an extra-adrenal paraganglioma and achieving a successful biochemical diagnosis.

Complete surgical resection remains the cornerstone therapy for localized BPGL, with partial cystectomy representing the most commonly employed surgical approach, as it preserves bladder function (5). Transurethral resection (TURBT) is generally not recommended as a definitive treatment because of the significant risks of incomplete resection, recurrence, and severe intraoperative complications, notably catecholamine-induced hypertensive crises (24). Regardless of the surgical technique chosen, meticulous preoperative preparation is paramount for ensuring perioperative safety. This primarily involves initiating alpha-adrenergic blockade (e.g., phenoxybenzamine) at least 7 – 14 days prior to surgery, combined with adequate volume expansion to control blood pressure and restore intravascular volume, thereby minimizing cardiovascular risks (6). Consequently, a correct preoperative diagnosis is vital, since over 95% of patients without an established diagnosis of BPGL do not receive pre-treatment with alpha-adrenergic antagonists, which increases their risk of intraoperative cardiovascular events (11). In the present case, the patient underwent laparoscopic partial cystectomy after thorough preparation. Despite this optimization, a transient hypertensive surge occurred during tumor resection but was promptly managed, allowing complete tumor excision. This outcome highlights the importance of comprehensive preoperative preparation, vigilant intraoperative monitoring, and coordinated multidisciplinary management.

Genetic factors significantly contribute to PPGL development, as pathogenic germline mutations are found in up to 40% of the patients. Additionally, researchers have linked these tumors to at least 12 distinct genetic syndromes (25). Consequently, genetic counseling and testing are recommended for all diagnosed patients to assess the personal risk for related neoplasms (such as RCC or GIST, particularly with SDHx mutations) and aid family screening (21). While most BPGLs behave benignly, approximately 13 - 15% exhibit malignant potential, for which reliable pathological predictors are currently lacking (3). Therefore, lifelong postoperative surveillance, typically involving annual biochemical monitoring supplemented by imaging as indicated, is advised for all patients with PPGL to detect recurrence or metastasis (6). Although the patient presented here declined genetic testing and showed a favorable short-term outcome, continued long-term monitoring remains essential because of the inherent risks associated with BPGL.

## Conclusions

In conclusion, this report describes an incidentally discovered BPGL in which ABPM confirmed a link between long-standing paroxysmal hypertension and micturition. This case underscores the diagnostic challenges of BPGL; its extreme rarity often leads to low clinical suspicion, and its clinical presentation is heterogeneous, with classic micturition-related symptoms affecting only a subset and frequently showing variable timing relative to voiding, which can obscure the identification of the critical trigger. Therefore, for patients presenting with unexplained paroxysmal hypertension, supplementing a detailed and comprehensive medical history with ancillary investigations, such as ABPM or AECG, to specifically identify key trigger factors is crucial for accurate diagnosis, helping avert misdiagnosis leading to inappropriate surgical interventions and adverse outcomes. Furthermore, for patients diagnosed with BPGL, thorough preoperative alpha-adrenergic blockade and collaborative multidisciplinary team management are paramount for ensuring safe and effective surgical treatment. Finally, potential malignancy and strong genetic factors necessitate genetic counseling, testing, and lifelong surveillance for every affected individual.

## Data Availability

The original contributions presented in the study are included in the article/supplementary material. Further inquiries can be directed to the corresponding author.
